# Supplementary material for: Biochemical and genetic analyses of the oomycete Pythium insidiosum provide new insights into clinical identification and urease-based evolution of metabolism-related traits
Source: PeerJ. 2018 Jun 5;6:e4821. doi: 10.7717/peerj.4821 (PMC5993020; doi:10.7717/peerj.4821)
Supplement: Supplemental Information 2 — Sequences of the putative urease genes of P. insidiosum identified in the genomes of P. insidiosum strains Pi-S (accession number, LC317047 for Ure1), Pi07 (accession number LC325168 for Ure1), and Pi45 (LC325169 for Ure1A, and LC325170 for Ure1B) have been submitted to the DDBJ database. [file peerj-06-4821-s002.docx]

>Strain Pi-S_(accession_LC317047)

CCGCGCGAAGAAGACCATCTCGTGCTTCACGCCGCCGGCGTCCTCGCGCAGAAGCGGCTGGCGCGTGGGCTGCGGCTCAACTACACGGAGAGCGTCGCGCTGCTCGCGACACAAGTGCTCGAGTTCATTCGCGATGGCAAAAAGCGTCGCCGAGCTCATGGCGCTCGGGACGCAGCTGCTCGGGCGCCGCCACGTGCTGGACGGAGTAGCCGAGATGCTGGACGAAGTGCAGGTCGAAGGCACGTTTCCCGACGGTACCAAGCTCGTGACGGTGCATCACCCGATCGCGACGATGGATGGAAACCTCGAGCTCGCGCTCTACGGATCGTTTCTGCCGGTTCCGTCCGCCGACTGCTTTCCGTTGCCGGCGTCCGTGGCAGTAACGAGGCTGGCAGAGGCGCCTGGCGCGGTGCTCACCGCAGACGAGGAGCTCGTGCTCAACGGCAGTCGGCAGCCACGCGCGCTGGAGATCACGAACCTGACGGATCGACCCATTCAGGTGGGCAGCCACTACCACCTCATTGAGGCGAACCCGTACCTTGAGATGGATCGCAAGCGCGCGTACGGCTACCGCCTCAACATTCCGTCTGGCACTGCCGTTCGTTTCGAGCCTGGGGATCGCAAGACCGTGTCGACGGTGCCGATCGGCGGCAATCGGGTCATCACGGGCGGCAACAACCTCGCGAGCGGCGTGGTGGACGTCGCCGCCGCGGACGCGATCGTGGCGAAGGCGGTGGAGAAGGGCTTCCACCATGCGCCGATGGTCGTGTCGTCTGAGGAAGACGCGCGCAATGCGATCGCCATGGTCTGCCGGATGCCGCGCAGTGTGTATGCGCAGACGTACGGCCCCACGACTGGCGACGTCGTGCGGCTCGGCGACATGGAGTTGTACGTGACGATCGAGCGCGACATGACTGTCTACGGCGACGAGTGCAAGTTTGGCGGTGGCAAAGTGTTGCGCGAAGGCATGGGCCAAGCCTCGGGCCTCGCGGCAGGCCAAGTGCTGGATACGATCATCACGAACGCGTTGATCCTCGACTACACGGGCATCTACAAGGCCGACATCGGCATCAAGGACGGGCTCATCGCTGGCATCGGCAAAGGGGGCAATCCGGACGTGATGGACGGCGTGCTGCCCAACATGATTGTCGGCGTGAACACGGAGGTCATCGCGGGCGAGGGACTGATCGTGACTGCCGGTGGCATGGATGCTCACGTGCACTTCATCTGCCCTCAGCTCTGCACGGAAGCACTCGCGAGCGGACTCACGACGCTCGTCGGCGGTGGCACAGGCCCCGCGACCGGCACCAACGCGACGACGTGCACGCCTGGACCCGCGCACATGAAGCTCATGCTCCAGGCGACGGACGTGATCCCGATCAACATTGGGCTCACGGGCAAAGGCAACACCTCGATGCCGGAGGGACTTCAGGACATCATTGACGCGGGTGCCGTGGGCATGAAGCTGCATGAAGACTGGGGCACGACGCCCGCCGCGATCGATACATGCCTCACTGTCGCCGAGGAGAACGATGTGCAGGTGACGATCCACACGGACACGCTGAACGAGTCTTGCTGCGTCGAGCACACGATCGCGGCGTTCAAAGGACGCACGATCCACACGTATCACAGCGAAGGCGCCGGCGGAGGCCACGCGCCCGACATCATCTCTGTCTGTGGCGTGCCCAACGTGCTGCCGTCGTCCACGAATCCCACGCGGCCGTACACGGTCAACACGATCGACGAGCATGTGGATATGCTGGTACGTCTATCCGGCCGCCCGTCCGTCAGTCATTTGATCACGATGAACTGGACATTGACGCTGCTCTGTGCGTAGATGGTGTGCCACCACTTGGACAAGAACATCGCGGAAGACGTCGCGTTCGCCGAGTCTCGCATCCGTGGCGAGACGATCGCCGCCGAAGACCTGCTGCACGACATGGGCGCCATCAGCATCATCTCGTCCGACTCGCAGGCCATGGGCCGCATCGGCGAAGTCGTCACGCGCACGTGGCAGACCGCTGACAAGATGAAGCGCGAGCGAGGCCCGTTGCCCGAGGACGCGGCGGACACAGCGCCGCGTGGCAAGACGCTCGCCGGCGCCGTGGTCTGCGACAACTTCCGCGTGAAGCGGTACGTCGCCAAGTACACGATCAATCCTGCGATCGCGCACGGCATGGCGCACCTGATCGGGTCCGTTGAGCCCGCCAAGCTCGCGGACCTGTGCCTCTGGCGCCCGGCGTTCTTCGCGAGCAAGCCCGAGATGGTGCTCAAGGGAGGCGTGATCGTGTTCGCGCAGATGGGCGACCCGAACGCCTCGATCCCGACGCCGCAGCCTGTCAAGATGCGGCCCATGTTCGGCTCGTTGGGCGCGGCCGTCGGCGCGTCGTCGGTCGCGTTTGTGAGTCGCAGCTGCGTCGACAAGCAGATCGCGCAGAGCTANGGACTCCGGAAGCGCATCGAGGCGGTGCGTCGCTGCCGCGACGTCACCAAGCGGGACATGAAGCTCAANGACGCGCTGCCGCAGCTCCGTGTGGACCCCGAGACGTACCGTGTGACGGCCGACGGCGAGTGGCTCACGTGCGCGCCGTCCAAGGAGCTGCCGCTGGCGCAGCGCTTCTTCCTGTTCTAA

>StrainPi07_(Accession_LC325168)

CCCCGCGAAGAAGACCATCTCGTGCTGCACGCCGCTGGCGTTCTCGCGCAGAAGCGGCTCGCGCGCGGACTGCGCCTCAACTACACGGAGAGCGTGGCGCTGCTCGCGACGCAGGTGCTCGAGTTCATCCGCGATGGCAAAAGCGTCGCTGAGCTCATGGCGCTCGGGACGCAGCTCCTCGGGCGCCGCCACGTGCTGGACGGCGTTGCCGAGATGCTGGACGAAGTGCAGGTCGAAGGCACGTTTCCCGACGGTACCAAGCTTGTGACGGTGCATCACCCGATCGCAACGATGGATGGGAATCTCGAGCTCGCGCTGTACGGCTCATTTCTGCCGGTTCCGTCAGCCGACTGCTTTCCGCTGCCAGATTCGGCGGCAGTCACGACGCTGATGGAGGCGCCTGGCGCGGTGCTCACCGCAGATGAGGAGCTCGTGCTTAACGGCGGTCGGCAGCCACGCGCGCTCCAGATCACGAACCTGACGGATCGACCCATTCAGGTGGGCAGCCACTACCACCTCATTGAGGCGAACCCGTATCTCGAGATGGACCGTAAGCGCGCGTACGGCTACCGACTGAACATTCCGTCGGGCACAGCCGTTCGCTTCGAGCCGGGGGATCGCAAGACCGTGTCGACGGTGCCGATCGGCGGCAACCGAGTCATCACTGGCGGCAACAACCTCGCGAGCGGCGCTGTTGACGCCGCTGCAGCGGACGCGATCGTGGCAAAGGCCGTGGAGAAGGGATTCCATCACGCGCCGATGGTCGTGTCGCCAGAGGAAGATGCGCGCAATGCGATCGCGATGGTCTGCAGAATGCCGCGCAGTGTGTACGCTCAGACGTACGGCCCCACGACGGGCGACGTCGTGCGTCTCGGCGACATGGAACTGTACGTGACGATCGAGCGCGACATGACGGTCTACGGCGACGAGTGCAAATTTGGCGGAGGCAAAGTGCTGCGAGAAGGCATGGGCCAAGCGTCGGGGCTCGCGGCGTCTCAAGTGCTGGACACGATCATCACGAACGCACTGATCCTCGACTACACGGGGATCTACAAGGCGGACATCGGCATCAAGGACGGACTCATCGCTGGGATCGGCAAAGGAGGAAATCCGGACGTGATGGACGGCGTCATGCCCAACATGATCGTCGGTGTGAACACGGAGGTCATCGCGGGTGAAGGGCTCATCGTCACTGCCGGTGGCATGGACGCGCACGTGCACTTCATCTGCCCTCAGCTGTGCACGGAAGCACTCGCGAGCGGACTCACGACACTCGTTGGCGGAGGCACAGGCCCCGCGACCGGCACCAACGCGACGACGTGCACCCCCGGACCCGCGCACATGAAGCTCATGCTGCAGGCGACAGACGTGATCCCGATCAACATTGGGCTCACGGGCAAAGGCAACACTTCGGTGCCAGATGGACTTCAAGACATCATCGACGCAGGCGCCGTGGGGATGAAGCTGCATGAGGATTGGGGTACGACGCCCGCCGCGATCGACACGTGCCTGACTGTCGCGGAGGAGAACGACGTGCAGGTGACGATCCACACAGACACGCTAAACGAGTCTTGCTGTGTGGAACACACGATCGCGGCGTTCAAGGGGCGCACGATCCACACGTACCACAGCGAAGGAGCCGGCGGAGGCCACGCGCCCGACATCATCTCTGTCTGTGGCGTGCCCAATGTGCTGCCGTCGTCTACGAACCCCACGCGGCCGTACACGGTCAACACCATCGACGAGCACGTGGATATGCTGGTACGTCGTGTCACGATCATTCCCTGTATGTGGATGTGCTGATGGTGTATTGCGTAGATGGTGTGCCATCATTTGGACAAAAACATCGCAGAGGACGTCGCGTTCGCCGAGTCTCGCATCCGTGGTGAGACGATCGCCGCTGAAGATCTGCTGCATGACATGGGCGCCATCAGCATCATCTCGTCCGACTCGCAGGCCATGGGCCGCATCGGCGAAGTCGTGACGCGCACGTGGCAGACCGCTGACAAGATGAAGCGCGAGCGAGGGCCATTGCCCGAGGACGCGGCGGACACGGCGCCGCGCGGCAAGACGCTCACCGGCGCCATGGTCTGCGACAACTTCCGCGTCAAGCGATACGTCGCCAAGTACACGATCAACCCTGCGATCGCGCACGGCATGGCGCACCTGATCGGGTCGGTCGAGCCTGCCAAGCTCGCTGATCTGTGTCTGTGGCGGCCGGCGTTCTTTGCGAGCAAGCCCGAGATGGTGCTCAAGGGAGGCGTGATCGTGTTCGCGCAGATGGGCGACCCGAACGCCTCGATTCCGACGCCGCAGCCCGTGAAGATGCGGCCCATGTTCGGCTCGTTGGGCGCAGCCGTCGGCGCGTCGTCGGTGGCGTTCGTGAGTCGCAGCTGCGTCGACAAGAAGATCGCGGAGAGCTACGGACTCCAGAAGCGCATTGAGGCAGTGCGTCGCTGCCGCGACGTGACCAAGCGCGACATGAAGCTCAACGACGCGCTGCCGCAACTGCGCGTGGACCCCGAGACGTACCGTGTGACGGCGGACGGCGTGTGGCTGACGTGCTCGCCGTCCAAGGAGCTGCCGTTGGCGCAGCGCTTCTTCCTGTTCTAGGCGATGCACGTAAATGTACAGTCGTGTTGGTGTTGCTAGTCGGCA

>SrainPi45_(Accession_LC325169)

CCCCGCGAAGAAGACCACCTCTTGCTCCACAGCGCTGGCGTCTTGGCGCAGAAGCGTCTCGCGCGCGGATTACGCCTTAACTACACCGAGAGCGTGGCGCTGCTCGCGACCCAAGTGCTCGAGTTCATTCGAGACGGCAAAAGCGTTGCAGAGCTCATGTCACTCGGCACACAGCTGCTTGGGCGCCGCCACGTGCTAGACGGAGTGAGCGACATGCTTGATGAAGTGCAAGTCGAAGGCACGTTCCCCGACGGCACCAAGCTCGTGACGATCCACCACCCAATCGCCACAATGGACGGGAATCTCGAGCTCGCGCTTTATGGATCGTTTCTTCCTGTTCCGCGCGCTGACTGTTTTCCGCTGCCTGAGGCAGCAGTGGCCACACAGCTCGTGCAGGCACCTGGTGGTGTGTTAACAGTGAACGACGAGCTTGTACTCAACGCCTTTCGTAAGCCACGCGCACTGCAGATCACAAACTTGACGGATCGCCCCATTCAAGTTGGAAGCCATTACCACCTCATCGAAGCGAACCCGTACCTCGAGATGGATCGGAAGCGAGCATACGGCTACCGCCTCAACATCCCATCCGGCACCGCCGTCCGATTCGAGCCTGGCGACCAGAAGACCGTTTCGACTATTCCGATAGGTGGCAACCGTGTCATCACGGGCGGAAACAACCTCGCAAGTGGTGTTGTAGACGAGGCCGTGGCGGATGATATCGTCGCGAAGGCGGTGGAGAAGGGCTTTCACCACAGGCCGATGGTCGTTTCTCCAGAGGAGGAAGCTCGCAACGCTGTCGCGATGATCTGCAGAATGCCACGCAGTGTGTACGCTCAGACGTACGGCCCGACCACTGGTGACGTCGTGCGACTCGGCGACATGGAGCTGTACGTGACTGTCGAGCGGGACTTGACGGTATATGGAGATGAGTGCAAATTCGGTGGAGGCAAAGTCCTCCGAGAAGGCATGGGGCAAGCTTCCGGTCTGATGGCGGCTCAAGTGCTTGATACGATCATCACCAACGCTCTCATTATCGACTACACCGGGATCTACAAGGCAGACATTGGCATCAAGGATGGACTCATCGCTGGTATTGGAAAGGGAGGTAATCCTGATGTCATGGATGGAGTCGCGCCCAACATGATTGTCGGTGTGAACACGGAGGTGATCGCGGGTGAGGGCCTGATTGTCACTGCTGGCGGCATGGATGCGCATGTACACTTCATCTGCCCCCAGCTGTGCACGGAGGCTCTCGCGAGCGGCTTGACAACGCTAGTCGGTGGCGGCTCCGGTCCGGCGACTGGCACCAACGCGACGACGTGCACGCCAGGGCCAGGGCATATGAAGCTCATGCTGCAGGCGACGGACGTGATCCCAATGAATATAGGGCTTACGGGCAAGGGCAACACCTCGATGCCAGAGGGCCTTCAGGACATTATTGACGCAGGCGCCGTGGGCATGAAACTCCATGAAGACTGGGGCACCACACCCGCCGCAATTGACACATGCCTGACGGTTGCAGAGGAGAACGATGTGCAGGTGACGATCCACACAGACACGTTGAACGAGTCTTGCTGTGTGGAACACACGATCGCCGCGTTTAAAGGGCGCACGATCCACACGTACCACAGCGAAGGCGCTGGGGGAGGCCATGCACCAGATATCATCTCTGTCTGCGGCGTTGCCAACGTGCTTCCTTCGTCAACCAATCCGACGCGGCCCTACACTGTCAACACCATCGACGAGCACGTGGATATGCTGGTACGTATCATCATCTCTGTCGAAAGGGAAGTTCATTGGCTCCCCTCTCACGACTTTGTGCTCACCGTAGATGGTTTGCCACCATTTGGACAAGAACATCGCTGAGGACGTGGCGTTCGCGGAGTCGCGAATCCGCGGCGAGACAATCGCTGCGGAGGACATACTCCACGACATGGGAGCCATCAGTATCATTTCATCCGACTCTCAAGCCATGGGCCGAATCGGCGAGGTCGTGACCCGCACTTGGCAAACTGCCGACAAAATGAAGCGCGAGCGAGGCCCGTTGCCTGAGGACAAAGAGGACTCGGCTCCGCGCGGGAAGACACTGAGCGGCGACGTCGTCTGCGACAACTTCCGCGTCAAGCGCTACGTCGCCAAGTACACGATCAACCCAGCGATCGCGCATGGCATGGCACACCTCATCGGGTCGATCGAGCCCTCGAAGATGGCAGATCTGTGCTTGTGGCATCCCGCGTTTTTCGGTAGCAAGCCCGAGATGGTCATCAAGGGCGGCGCCATCGTGGTCGCGCAGATGGGCGACCCGAACGCCTCGATTCCGACCCCACAGCCCGTGAAAATGCGGCCCATGTTTGGCGCAATGGGGGCTGCCGTGGGACCGACGTCGGTCGCCTTTGTAAGCCGCAGCTGCGTCGACAAGAAGATCGCAGAGGCGTACGGACTCCAGAAGCGCATCGAGGCCGTGCGTCGCTGCCGGGGCGTCACCAAGCGAGACATGAAGCTCAACGACGCGCTGCCGCAGGTCCGCGTGGACCCCGAGACGTACCGCGTGACGGCGGACGGCGTCTGGCTCACATGCGGGCCGTCGCAGCAGCTCCCGTTGGCGCAGCGCTACTTTTTGTTTTAGGAATATGCATCCGTTGTTTGTCCGTGAAGAGT

>StrainPi45_(Accession_LC325170)

CCCCGCGAAGAAGACCACCTCTTGCTCCACAGCGCTGGCGTTTTGGCGCAGAAGCGTCTCGCGCGCGGATTGCGCCTCAACTACACCGAGAGCGTGGCGCTGCTCGCGACCCAAATGCTCGAGTTCATTCGAGACGGCAAAAGCGTTGCAGAGCTCATGTCACTCGGCACGCAGCTGCTGGGGCGTCGCCACGTGCTAGACGGAGTGGGCGACATGCTTGACGAAGTGCAGGTCGAAGGCACGTTCCCCGATGGCACCAAGCTCGTGACGATCCACCACCCAATCGCCACAATGGACGGGAATCTCGAGCTCGCACTTTATGGATCGTTTCTTCCTGTTCCGCTCGCTGACTGTTTTCCGCTGCCTGAGGCAGCAGTCGCTACACAGCTCGTGCAGGCACCTGGTGGTGTGCTAACAGTGAACGACGAGCTTGTACTCAACGCCTCTCGTAAGCCGCGCGCGCTGCAGATCACAAACTTGACAGATCGCCCCATTCAAGTTGGAAGCCATTACCACCTCATCGAAGCGAACCCGTACCTCGAGATGGATAGGAAGAGGGCATACGGCTATCGCCTCAACATCCCATCCGGTACCGCCGTCCGATTCGAGCCTGGCGATCGGAAGACCGTGTCGACTATTCCAATCGGTGGCAACCGTGTCATCACGGGCGGAAACAACCTCGCAAGTGGTGTTGTAGATGAGGCCGCGGCGGATGGTATCGTCGCTAAGGCGGTGGAGAAGGGTTTTCACCACAAGCCGATGGTCGTGTCACCGGAAGAGGAAGCTCGTAACGCTGTCGCGATGATCTGCAGAATGCCACGCAGTGTGTACGCTCAGACGTACGGCCCGACCACTGGTGACGTCGTGCGACTCGGTGACATGGAGCTGTACGTGACTATCGAGCGAGACTTGACGGTGTACGGAGATGAGTGCAAGTTCGGTGGCGGCAAAGTCCTCCGAGAAGGCATGGGACAAGCTTCAGGTCTGATGGCGGCTCAGGTGCTTGATACAATCATCACCAACGCTCTCATTATCGACTACACAGGGATCTACAAAGCAGATATCGGCATCAAGGATGGATTTATCGCTGGTATTGGGAAGGGAGGCAATCCTGATGTCATGGATGGAGTCGTGCCAAACATGATTGTCGGTGTGAACACGGAGGTGATCGCGGGCGAGGGCCTCATTGTCACTGCTGGTGGCATGGATGCGCATGTACACTTCATCTGCCCCCAGCTGTGCACGGAGGCGCTCGCGAGCGGCTTGACAACACTAGTGGGTGGCGGCTCCGGACCGGCGACCGGCACCAACGCGACGACGTGCACGCCAGGGCCAGCGCATATGAAGCTGATGCTGCAGGCGACTGACGTGATCCCAATGAATATCGGGCTTACGGGCAAGGGCAATACCTCCATGCCAGAGGGTCTTCAGGACATTATTGACGCAGGCGCCGTGGGCATGAAACTCCATGAAGACTGGGGCACCACACCCGCCGCGATTGACACATGCCTGACGGTTGCAGAGGAGAATGATGTGCAGGTGACGATCCACACAGACACGTTGAACGAGTCTTGTTGCGTGGAACACACGATCGCCGCGTTCAAAGGGCGCACGATCCACACGTACCACAGCGAAGGCGCTGGGGGAGGCCATGCACCAGATATCATCTCGGTCTGCGGCGTTGCTAACGTGCTTCCTTCGTCCACCAATCCGACGCGACCCTACACTGTCAACACCATCGACGAGCACGTGGATATGCTGGTACGTATCTCCATCGCTGTCGACAGGAAGCTCACTGGTTACCCTCTCACGACGTTGTGCGCGCCGTAGATGGTCTGCCACCATTTGGACAAGAACATCGCTGAAGACGTGGCGTTCGCGGAGTCGCGTATCCGCGGCGAGACGATCGCTGCGGAGGACTTACTCCACGACATGGGGGCCATCAGCATCATTTCATCCGACTCGCAAGCGATGGGCCGAATCGGCGAGGTCGTGACCCGCACTTGGCAGACTGCCGACAAGATGAAGCGTGAGCGAGGCCCGTTGCCCGAGGACAGAGAGGACTCGGCGNNNNNNNNNNCTGAGCGGCGACGTCGTCTGCGACAACTTCCGCGTCAAGCGCTACGTCGCCAAGTACACGATCAACCCAGCGATCGCGCACGGCATGGCACACCTCATCGGGTCGATCGAGCCCTCGAAGATGGCAGATCTGTGCCTGTGGCATCCGGCGTTTTTCGGTAGCAAGCCCGAGATGGTCATCAAGGGCGGCGCCATCGTAGTCGCGCAGATGGGCGACCCGAACGCCTCGATTCCGACCCCACAGCCCGTGAAAATGCGGCCCATGTTCGGCGCAATGGGGGCTGCCGTCGGGCCAACGTCGGTCGCCTTTGTGAGCCGCAGCTGCGTCGACAAGAAGATCGCAGAGGCGTACGGACTCCAGAAGCGCATCGAGGCCGTGCGTCGCTGCCGGGGCGTCACCAAGCGCGACATGAAGCTCAACGACGCGCTGCCGCAGATCCGCGTAGACCCAGAGACGTACCGCGTGACGGCGGACGGCGTCTGGCTCACGTGCGGGCCGTCGCAGCAGCTCCCGTTGGCGCAGCGCTACTTTTTGTTTTAGGAATATGCATCCGCTTGTGTGATTTGTCCGTGAAGAGT
